# Effect of Personalized Messages Sent by a Health System’s Patient Portal on Influenza Vaccination Rates: a Randomized Clinical Trial

**DOI:** 10.1007/s11606-021-07023-w

**Published:** 2021-09-01

**Authors:** Peter G. Szilagyi, Christina S. Albertin, Alejandra Casillas, Rebecca Valderrama, O. Kenrik Duru, Michael K Ong, Sitaram Vangala, Chi-Hong Tseng, Sharon G. Humiston, Sharon Evans, Michael Sloyan, Jonathan E. Bogard, Craig R. Fox, Carlos Lerner

**Affiliations:** 1 Department of Pediatrics, UCLA Mattel Children’s Hospital, University of California at Los Angeles, Los Angeles, CA USA; 2 Department of Medicine, David Geffen School of Medicine, University of California, Los Angeles, Los Angeles, CA USA; 3 VA Greater Los Angeles Healthcare System, Los Angeles, CA USA; 4 Department of Health Policy and Management, Fielding School of Public Health, University of California, Los Angeles, Los Angeles, CA USA; 5 Department of Medicine Statistics Core, David Geffen School of Medicine, University of California, Los Angeles, Los Angeles, CA USA; 6 Department of Pediatrics, Children’s Mercy, Kansas City, MO USA; 7 Department of Information Services and Solutions, UCLA Health System, Los Angeles, CA USA; 8 UCLA Anderson School of Management, Los Angeles, CA USA; 9 Department of Psychology, UCLA, Los Angeles, CA USA

## Abstract

**Background:**

Adult influenza vaccination rates are low. Tailored patient reminders might raise rates.

**Objective:**

Evaluate impact of a health system’s patient portal reminders: (1) tailored to patient characteristics and (2) incorporating behavioral science strategies, on influenza vaccination rates among adults.

**Design:**

Pragmatic 6-arm randomized trial across a health system during the 2019–2020 influenza vaccination season. The setting was one large health system—53 adult primary care practices.

**Participants:**

All adult patients who used the patient portal within 12 months, stratified by the following: young adults (18–64 years, without diabetes), older adults (≥65 years, without diabetes), and those with diabetes (≥18 years).

**Interventions:**

Patients were randomized within strata to either (1) pre-commitment reminder alone (1 message, mid-October), (2) pre-commitment + loss frame messages, (3) pre-commitment + gain frame messages, (4) loss frame messages alone, (5) gain frame messages alone, or (6) standard of care control. Patients in the pre-commitment group were sent a message in mid-October, asking if they planned on getting an influenza vaccination. Patients in loss or gain frame groups were sent up to 3 portal reminders (late October, November, and December, if no documented influenza vaccination in the EHR) about importance and safety of influenza vaccine.

**Main Measures:**

Receipt of 1 influenza vaccine from 10/01/2019 to 03/31/2020.

**Key Results:**

196,486 patients (145,166 young adults, 29,795 older adults, 21,525 adults with diabetes) were randomized. Influenza vaccination rates were as follows: for young adults 36.8%, for older adults 55.6%, and for diabetics 60.6%. On unadjusted and adjusted (for age, gender, insurance, race, ethnicity, and prior influenza vaccine history) analyses, influenza vaccination rates were not statistically different for any study group versus control.

**Conclusions:**

Patient reminders sent by a health system’s patient portal that were tailored to patient demographics (young adults, older adults, diabetes) and that incorporated two behavioral economic messaging strategies (pre-commitment and loss/gain framing) were not effective in raising influenza vaccination rates.

**Trial Registration:**

This trial was registered with ClinicalTrials.gov (NCT04110314).

**Supplementary Information:**

The online version contains supplementary material available at 10.1007/s11606-021-07023-w.

## INTRODUCTION

Influenza causes substantial morbidity and mortality among adults.^1,2^ The Advisory Committee on Immunization Practices (ACIP) recommends annual influenza vaccination for all US adults,^1,2^ and the US Healthy People 2030 goal is >70% influenza vaccination rates.^3^ However, in 2019–2020 (before the pandemic), US vaccination coverage for the adults was as follows: 18–49 years (38.4%), 50–64 years (50.6%), and 65+ years (69.8%).^4^

One strategy to raise influenza vaccination rates is vaccination reminders to patients—usually via telephone, autodialer, or mail; this is supported by a recent Cochrane review.^5^ Although recommended by the Task Force on Community Preventive Services for vaccinations,^6^ few primary care practices send reminders.^7,8^ Experts recommend centralized reminders sent by health systems. Two studies testing reminders from state immunization information systems for children found variable impact;^9,10^ authors speculated that reminders from patients’ primary care providers might have greater impact. Little is known about vaccination reminders for adults, with few recent studies^5^ in today’s era of vaccine hesitancy.^11–13^

Many electronic health records (EHRs) have patient portals: secure, Internet sites, and mobile applications allowing patients and healthcare providers to communicate.^14^ Portals are used widely,^14^ particularly with telehealth.^15^ A 2014–2015 randomized trial evaluating patient portal plus interactive voice response reminders found only 1% improvement in influenza vaccination rates in a population with extremely low baseline coverage.^16^ We evaluated the impact of one, two, or three generic portal reminders and found only minimal impact on influenza vaccination rates among adults and children for the 2018–2019 vaccination season.^17^ It is unclear whether the negative findings were due to suboptimal messaging or to limited impact of portal reminders themselves.

Tailored messages targeting health beliefs can improve some health behaviors.^18^ Concerns about influenza vaccine include vaccine safety and efficacy.^13,19^ Also, healthy young adults might feel less vulnerable to influenza disease and those with chronic conditions more vulnerable.^13^ Thus, reminder messages tailored by age (i.e., young adults, older adults) and chronic conditions (e.g., diabetes) might improve their effectiveness.

For this study, we expected to increase vaccination uptake by utilizing principles from social psychology^20^ and behavioral economics^21,22^ by testing two strategies: pre-commitment and gain loss framing. Pre-commitment is a strategy in which people are asked to commit today to engage in a future target behavior, harnessing people’s desire to act consistently with public statements and their prior active choices.^23–26^ We expected that having patients pre-commit to their doctor that they will obtain the influenza vaccine should increase their follow-through when later encouraged to do so.

Gain loss framing is a strategy in which a message is described as what a person has to gain versus to lose by taking a particular action. A cornerstone of behavioral economics is the observation that decisions are influenced by whether outcomes are framed as losses or gains, even when options are objectively equivalent.^21^ Several studies have suggested that framing influences decisions by doctors and patients.^27–29^ Reversing losses was found to provide stronger motivation than comparable gains for HPV vaccination in one study^28^ (although not in another).^30^ A review of loss/gain framing for vaccinations noted studies on HPV vaccine with a suggestion that parents might be more persuaded by loss framed messaging.^31^ Based on studies on HPV vaccination, we expected that presenting messages highlighting costs of not getting an influenza vaccine would lead to higher vaccination rates than messages highlighting the benefits of vaccination.

We performed a 6-­arm randomized clinical trial comparing the impact of (1) pre-commitment versus no pre-commitment and (2) negative or positive gain frame messaging, on receipt of influenza vaccination for three patient groups within a large health system: young adults (18–64 years), older adults (65+ years), and patients with diabetes (18+ years).

## METHODS

### Study Design

**Figure 1:**
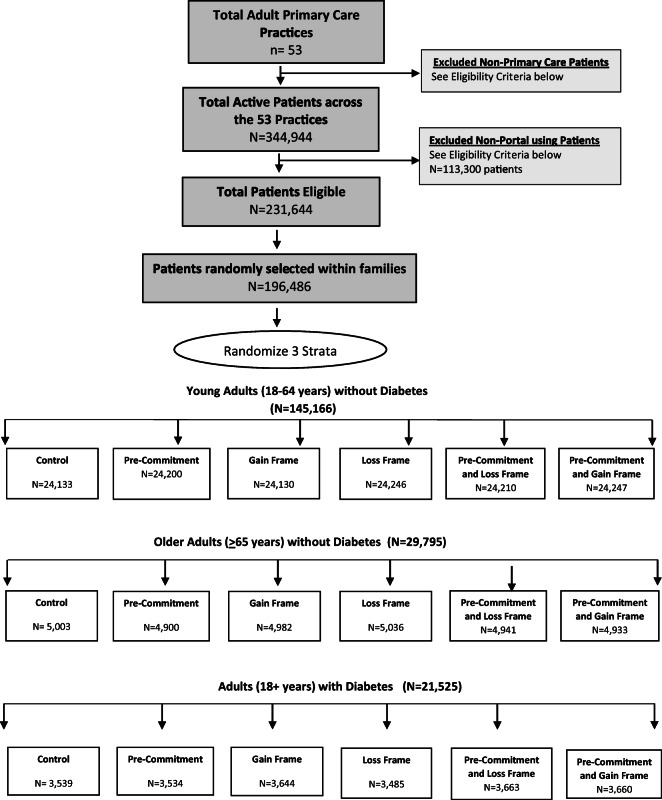
Consort diagram

The University of California, Los Angeles (UCLA) IRB approved the study with waiver of patient consent. Between 10/1/2019 and 3/31/2020 (pre-pandemic), we conducted a 6-arm RCT (Figure 1), randomizing patients to: control (no messages), pre-commitment letter only, pre-commitment letter plus loss framed reminders, pre-commitment letter plus gain framed reminders, loss framed reminders only, and gain framed reminders only. Pre-commitment groups received 1 pre-commitment message in mid-October. Loss or gain frame groups received up to 3 portal reminders—emphasizing the importance and safety of influenza vaccine—in late October, November, and December if no documented influenza vaccination. We conducted the trial across all 53 internal medicine, medicine-pediatric, and family medicine primary care practices at UCLA Health.

### Study Participants

All practices had the same *Epic*™ EHR and portal. First, we identified all primary care patients ≥18 years (Figure 1). The health system defines primary care patients by the following: ≥2 primary care provider (PCP) visits (by Evaluation and Management office codes) within 3 years or ≥1 PCP visit with preventive service codes within one year, or managed care patient assigned to UCLA Health (irrespective of visits). Second, we identified the primary care practice most recently visited within three years. Third, we grouped patients into family units with algorithms matching patient’s phone number, address, insurance member number, or patient guarantor ID. Fourth, we identified *active portal users* as patients or portal proxies (for elderly or disabled persons) who logged into the portal at least once in 12 months, not including the initial portal login ( 67% of primary care patients). Fifth, we stratified all patients from the same family by patient groups (*young adults* 18–64 years without diabetes, *older adults* ≥65 years without diabetes, adults ≥18 years with *diabetes* [including diabetes type 2] per SUPREME criteria^32^). Sixth, statisticians randomly selected one active portal-using index patient per family within each stratum, generating the denominator of potential subjects; other study personnel and healthcare providers were blinded to study allocation. We excluded patients who were not active portal users and family members of index subjects (for consistency with Cochrane criteria for meta-analyses).^5^

Study statisticians randomized index subjects to one of six study arms. Family members of the index subjects were sent identical portal reminders to prevent confusion; we analyzed data for index subjects.

### Portal Message Development

We framed portal messages following the Health Belief Model,^33^ our prior portal study,^17^ principles of health literacy,^34^ and behavioral economics.^20,35,22^ We first pre-tested several psychological and behavioral economic principles on Amazon’s Mechanical Turk platform (MTurk)^36^ (we could not test pre-commitment). We collected 3,896 US subjects, introduced the task, and randomly assigned them to one of 22 experimental arms—a control and a treatment message for 11 different psychological principles (Appendix 1 in the Supplementary Information). We asked five questions about the influence of particular messages on likelihood of influenza vaccination, constructed a composite measure of these five items, and asked how a message would change intention to vaccinate. We analyzed the impact of each method on vaccination intent, hypothetical change, and the composite measure. We found statistically significant and positive results for four psychological principles: *gains framing* (failure to vaccinate as a foregone gain rather than a loss), *scarcity appeals* (time is short to vaccinate), *commission framing* (choosing to vaccinate), and *authoritative messenger* (message from an authority).

Based upon these findings, we formally tested the impact of loss/gain messaging in the field because this principal is debated in the vaccination literature^31^ and our MTurk results ran counter to our a priori expectation. We also incorporated phrases emphasizing *scarcity*, *commission*, and *appeal to authority* in all portal messages, but did not test them formally since the design already had multiple study arms.

### Intervention

Study statisticians sent files to the health system’s EHR team, defining which portal message to send per round. Patients were sent by system-generated notification via email or text (per patients’ portal preference settings) that “A message from your doctor” was on the portal. Patients logged into the portal to read the message.

System-generated messages were in English, were at <7th grade reading level per Flesch-Kincaid analysis, and included PCPs’ names. All messages (Appendix 2 in the Supplementary Information) contained the following: (a) Dear “First Name”; (b) sentences following three behavioral principles, i.e.,^20,35^ *scarcity* (“time is running out to maximize the benefit of your flu vaccine”), *appeal to authority* (i.e.,“UCLA doctors … strongly recommend the flu vaccine each year for persons 65 years and older”), and *commission* (i.e., “Choosing to get vaccinated this season…”); (c) a link (and phone number) with “Call us to make an appointment or click here to request an appointment online”; and (d) a link to a website with information about influenza vaccine and video testimonials.

Loss framed messages stressed adverse consequences of choosing not to be vaccinated. Gain framed messages stressed positive consequences of choosing to get vaccinated.

Pre-commitment messages were sent in mid-October, asking patients if they planned to receive an influenza vaccine this season (Yes/No/Not sure). Identical portal reminders were sent in late October, November, and December (if loss or gain framed groups and if no vaccination was found by EHR 1 week before portal reminders).

### Measures

#### Patient Characteristics

Patient characteristics from the EHR (Table 1) are as follows: age, sex, insurance at latest primary care visit, race, ethnicity, and influenza vaccination within two years.

**Table 1:** Demographic Characteristics of the Study Sample by Intervention Strata^*^

	**Young adults 18–64yr without diabetes (*N*=145,166)**	**Older adults ≥65yr without diabetes (*N*=29,795)**	**Adults with diabetes (*N*=21,525)**
**Age**			
Mean (SD)	41.1 (11.9)	73.6 (7.2)	61.7 (14.9)
Median (Q1, Q3)	41.2 (32.5, 51.8)	71.8 (68.0, 77.2)	62.9 (52.2, 71.9)
Min, Max	18.0, 64.9	65.0, 106.7	18.1, 104.5
**Gender**			
Female	87,334 (60.2%)	17,059 (57.3%)	10,635 (49.4%)
Male	57,832 (39.8%)	12,736 (42.8%)	10,890 (50.6%)
**Primary insurer**^**†**^			
Private	139,188 (95.9%)	13,219 (44.4%)	14,602 (67.8%)
Public	3,682 (2.5%)	16,123 (54.1%)	6,586 (30.6%)
Other/unknown	2,296 (1.6%)	453 (1.5%)	337 (1.6%)
**Race**			
White	77,757 (53.6%)	21,836 (73.3%)	12,142 (56.4%)
Black/African-American	6,153 (4.2%)	1,192 (4.0%)	1,693 (7.9%)
Asian	15,039 (10.4%)	2,181 (7.3%)	2,933 (13.6%)
Other/multiple races/unknown	46,174 (31.8%)	4,581 (15.4%)	4,748 (22.1%)
**Ethnicity**			
Hispanic or Latinx	15,235 (10.5%)	1,621 (5.4%)	2,806 (13.0%)
Non-Hispanic/other/unknown	129,931 (89.5%)	28,174 (94.6%)	18,719 (87.0%)
**Influenza vaccine history**^**‡**^			
Prior vaccination	71,237 (49.1%)	22,367 (75.1%)	15,389 (71.5%)
No prior vaccination	73,929 (50.9%)	7,428 (24.9%)	6,136 (28.5%)

^*^For each of the 3 strata, patients were randomized to one of six groups: control, pre-commitment only, gain frame, loss frame, pre-commitment plus gain frame, and pre-commitment plus loss frame

^†^Public insurer included Medicaid, Medicare, and Tricare. If patients had Medicare + supplemental private Medigap coverage they were labeled as private

^‡^Notation of an influenza vaccination within the EHR over any one of the prior 2 influenza seasons

#### Influenza Vaccination Data

We obtained influenza vaccination date and location from the EHR if administered at any UCLA Health site. UCLA practitioners can enter vaccination records manually for outside vaccinations. This information was merged into the EHR along with influenza vaccination data from (1) SureScripts (pharmacy benefits manager), (2) California Immunization Registry, and (3) CareEverywhere (Epic’s information exchange application). Patients or proxies can also enter vaccination data via the portal. We integrated all data sources prior to analyses.

Since patients receiving our portal messages could enter vaccinations received elsewhere via a link; we included this information in a secondary outcome measure.

#### Outcome Measures

##### Primary Outcome

The primary study outcome was influenza vaccination between 10/01/2019 and 03/31/2020 (by EHR, after merging above sources). The primary analysis included all vaccinations *except* those self-reported by patients specifically in response to portal reminders as the control group did not have this opportunity for self-report, eliminating differential ascertainment. This analysis created a conservative bias since portal reminders may encourage patients to seek influenza vaccination at outside locations (e.g., workplace, pharmacies) not merged as above.

##### Subgroup Outcomes

These included influenza vaccination in the following: (1) pre-determined subgroups—sex, race/ethnicity (Black, White, Asian, Hispanic), primary insurer (public, private, other), and influenza vaccination within two years; (2) patients who self-reported in response to portal reminders if data were not in the EHR; (3) patients who opened ≥1 portal reminder; and (4) patients in the upper versus bottom half of overall portal usage.

##### Process Measures

We assessed the percentage of patients who opened the portal reminder letter and indicated a source of influenza vaccination obtained externally, and we checked whether each externally administered vaccination was already included in the EHR via the portal linkage processes.

### Power Calculation

We assessed power for the most conservative comparison—impact of tailored reminders among patients with diabetes. A sample size of ~7,200 patients per reminder letter arm provides 80% power to detect a 2.7 percentage point improvement in vaccination. This assumes a chi-squared test, control group rate of 50% (most conservative), and a significance level of 0.017 (3-fold Bonferroni correction for three main effects: loss frame, gain frame, and pre-commitment messages).

### Statistical Analysis

We report descriptive statistics for patient characteristics. Primary analyses compared vaccination rates between study arms using mixed effects Poisson regression with robust standard errors, stratifying patients into young adults, older adults, and patients with diabetes. Models included a fixed effect for reminder arm (loss frame versus gain frame versus no message), a fixed effect for pre-commitment arm (message versus no message), random practice effects, and adjustment for patient characteristics (age, gender, insurance, race, ethnicity, and prior vaccination). Secondary subgroup analyses were performed by fitting separate models for each subgroup.

For the primary analysis, a significance level of 0.017 was used. In all other analyses, we considered *p*-values below 0.05 as statistically significant.

As a secondary analysis, we used an instrumental variables approach to evaluate the effect of pre-commitment messages and loss/gain frame on subgroups who opened a portal message.

Statistical analyses were performed using SAS v. 9.4 (SAS Institute Inc., Cary, NC).

## RESULTS

### Practice and Patient Characteristics

We randomized 196,486 patients including young adults (*N*=145,166), older adults (*N*=29,795), and patients with diabetes (*N*=21,525) to one of six study groups (Figure 1). Most had private or Medicare insurance and were White (5–13% were Hispanic), and half to three-quarters had an influenza vaccination within 2 years (Table 1).

### Primary Outcome: Influenza Vaccination

Influenza vaccination rates were low—37% young adults, 55% older adults, 60% patients with diabetes. There were no substantive differences by either pre-commitment (Table 2) or message framing (loss vs gain, Table 3) within any of the three strata.

**Table 2:** Influenza Vaccination Rates Within Patient Strata (Young Adults 18–64 Years, Adults ≥ 65 Years, and Adults with Diabetes) and by *Pre-Commitment* (None Versus a Pre-Commitment Message). These Results *Exclude* Vaccinations Self-reported by Patients in Response to the Portal Influenza Reminders

**Pre-commitment (no/yes)**	**Young adults 18–64yr without diabetes**		**Older adults ≥65yr without diabetes**		**Adults with diabetes**	
	**No**	**Yes**	**No**	**Yes**	**No**	**Yes**
**All patients**	36.5%	37.0%	55.4%	55.9%	60.2%	60.9%
**Gender**						
Female	37.2%	37.5%	55.8%	55.8%	59.4%	60.9%
Male	35.5%	36.2%	54.8%	55.9%	61.0%	60.9%
**Primary insurer**						
Private	36.6%	37.0%	53.8%	54.7%	55.8%	57.1%
Public	37.1%	37.9%	56.5%	56.9%	70.4%	69.3%
Other/unknown	31.3%	32.9%	62.3%	52.4%	50.0%	63.2%
**Race**						
White	38.0%	38.5%	55.9%	56.3%	62.0%	63.5%
Black	27.6%	29.3%	45.2%	46.1%	52.0%	48.9%^*^
Asian	45.5%	45.0%	61.7%	62.2%	67.2%	67.8%
Other/multiple/unknown	32.3%	32.9%	52.8%	53.2%	54.0%	54.7%
**Ethnicity**						
Hispanic	35.3%	35.7%	51.0%	51.4%	58.1%	58.4%
Non-Hisp./unknown	36.7%	37.1%	55.6%	56.1%	60.5%	61.3%
**Vaccine history**						
None	16.4%	16.2%	17.1%	18.6%	19.1%	21.6%^*^
Prior vaccination^†^	57.5%^*^	58.6%	68.4%	67.9%	76.9%	76.3%

^*^p<0.05

^†^Prior influenza vaccination in the past 2 years

**Table 3:** Influenza Vaccination Rates by Strata (Adults 18–64 Years, Adults ≥65 Years, and Adults with Diabetes), and by *Reminder Framing* (None, Loss Frame, or Gain Frame). These Results *Exclude* Vaccinations Self-reported by Patients in Response to the Portal Influenza Reminders

	**Young adults 18–64yr without diabetes**			**Older adults ≥65yr without diabetes**			**Adults with diabetes**		
	**None**	**Loss frame**	**Gain frame**	**None**	**Loss frame**	**Gain frame**	**None**	**Loss frame**	**Gain frame**
**All patients**	36.7%	37.0%	36.6%	56.1%	55.2%	55.6%	60.9%	60.1%	60.7%
**Gender**									
Female	37.1%	37.6%	37.4%	55.9%	55.3%	56.2%	60.7%	59.6%	60.3%
Male	36.1%	36.1%	35.3%	33.1%	33.5%	33.4%	61.1%	60.6%	61.1%
**Primary insurer**									
Private	36.8%	37.0%	36.6%	53.8%	54.4%	54.4%	57.0%	55.6%	56.8%
Public	37.4%	38.3%	36.7%	57.9%	55.9%	56.4%	70.2%	70.1%	69.4%
Other/unknown	30.6%	34.5%	31.3%	56.3%	52.5%	63.8%	51.9%	59.2%	57.9%
**Race**									
White	38.1%	38.4%	38.2%	56.5%	55.9%	55.8%	62.6%	62.7%	62.9%
Black	26.8%	29.2%^*^	29.4%	42.3%	47.1%	47.5%	52.9%	48.6%	49.7%
Asian	45.7%	44.6%	45.5%	61.3%	61.7%	62.9%	68.5%	67.6%	66.6%
Other/multiple/unknown	32.7%	33.2%	31.8%^*^	54.7%	51.1%	53.3%	54.5%	53.4%	55.2%
**Ethnicity**									
Hispanic	36.1%	36.3%	34.2%^*^	49.9%	53.2%	50.4%	60.3%	56.3%^*^	58.1%
Non-Hisp./unknown	36.8%	37.1%	36.8%	56.4%	55.3%	55.9%	61.0%	60.7%	61.1%
**Vaccine history**									
None	15.9%	16.5%	16.4%	17.5%	18.5%	17.5%	19.5%	21.6%	20.1%
Prior vaccination^†^	58.4%	58.2%	57.5%^*^	68.6%	67.9%	68.0%	77.5%	76.0%	76.4%

^*^p<0.05

^†^Prior influenza vaccination in the past 2 years

### Secondary Analyses

#### Subgroups

There were no substantive differences in influenza vaccination rates by either pre-commitment (Table 2) or message framing (loss vs gain, Table 3) within any of the pre-defined demographic subgroups or by prior influenza vaccination.

### Multivariate Analyses

Table 4 shows risk ratios (95% confidence intervals) for both unadjusted and adjusted analyses, comparing the effect of pre-commitment and framing on influenza vaccination rates for each stratum. There was no statistically or clinically significant impact of either pre-commitment or loss/gain framing on influenza vaccination rates.

**Table 4:** Risk Ratios (95% Confidence Intervals)^*^ from Unadjusted and Adjusted Analyses, Comparing Loss/Gain Frame, Pre-Commitment (No/Yes), and Demographic Characteristics Within Each of the 3 Strata (Young Adults 18–64 years (Without Diabetes), Older Adults ≥65 Years (Without Diabetes), and Adults with Diabetes. These Results *Exclude* Vaccinations Self-reported by Patients in Response to the Portal Influenza Reminders

**Study arms and subgroups**	**Young adults 18–64yr without diabetes**		**Older adults ≥65yr without diabetes**		**Adults with diabetes**	
	**Unadjusted risk ratio**	**Adjusted risk ratio**	**Unadjusted risk ratio**	**Adjusted risk ratio**	**Unadjusted risk ratio**	**Adjusted risk ratio**
**Pre-commitment arm** *(Ref = none)*						
Pre-commitment message	1.01 (1.00, 1.03)	1.01 (1.00, 1.03)	1.01 (0.99, 1.03)	1.00 (0.99, 1.01)	1.01 (0.99, 1.03)	1.01 (0.99, 1.03)
**Reminder arm** *(Ref = no reminders)*						
Loss frame	1.01 (0.99, 1.02)	1.01 (0.99, 1.02)	0.98 (0.96, 1.01)	1.00 (0.98, 1.02)	0.99 (0.97, 1.01)	0.99 (0.97, 1.02)
Gain frame	0.99 (0.98, 1.01)	0.99 (0.98, 1.01)	0.99 (0.97, 1.02)	0.99 (0.97, 1.02)	1.00 (0.97, 1.02)	0.99 (0.97, 1.01)
**Age (+1y)**	**1.01 (1.01, 1.01)**	**1.01 (1.01, 1.01)**	1.00 (1.00, 1.00)	**0.99 (0.99, 0.99)**	**1.01 (1.01, 1.01)**	**1.01 (1.00, 1.01)**
**Gender** *(Ref = male)*						
Female	**1.02 (1.00, 1.04)**	**1.02 (1.01, 1.04)**	1.01 (0.98, 1.04)	**1.03 (1.00, 1.05)**	**0.98 (0.95, 1.00)**	1.00 (0.98, 1.02)
**Primary insurer** *(Ref = private)*						
Public	0.99 (0.94, 1.05)	0.98 (0.94, 1.03)	**1.04 (1.02, 1.06)**	**1.05 (1.03, 1.07)**	**1.22 (1.19, 1.26)**	1.01 (1.00, 1.03)
Other/unknown	**0.85 (0.77, 0.94)**	0.93 (0.87, 1.00)	1.03 (0.95, 1.12)	1.04 (0.97, 1.12)	0.98 (0.91, 1.05)	0.96 (0.91, 1.02)
**Race** *(Ref = White)*						
Black/African-American	**0.73 (0.69, 0.76)**	**0.85 (0.81, 0.88)**	**0.83 (0.79, 0.87)**	**0.89 (0.85, 0.94)**	**0.79 (0.75, 0.83)**	**0.90 (0.87, 0.94)**
Asian	**1.13 (1.10, 1.15)**	**1.08 (1.06, 1.10)**	**1.11 (1.07, 1.14)**	**1.07 (1.04, 1.10)**	**1.05 (1.01, 1.09)**	**1.04 (1.01, 1.07)**
Other/multiple races/unk	**0.84 (0.82, 0.86)**	**0.94 (0.92, 0.96)**	**0.94 (0.91, 0.97)**	1.01 (0.98, 1.04)	**0.86 (0.83, 0.90)**	**0.96 (0.93, 0.98)**
**Ethnicity** *(Ref =Non-Hisp/other/unk)*						
Hispanic or Latinx	**0.96 (0.93, 0.99)**	1.01 (0.98, 1.03)	**0.92 (0.87, 0.97)**	**0.92 (0.88, 0.96)**	**0.95 (0.92, 0.99)**	1.02 (0.99, 1.05)
**Vaccine history** *(Ref = None)*						
Prior vaccination^†^	**3.53 (3.39, 3.67)**	**3.44 (3.31, 3.58)**	**3.84 (3.60, 4.09)**	**3.87 (3.63, 4.13)**	**3.76 (3.55, 3.98)**	**3.60 (3.40, 3.81)**

^*^The *p*-values for pre-commitment message and loss or gain frame use a significance threshold of 0.017; the rest use 0.05. All boldfaced cells have *p≤*0.01

^†^Prior influenza vaccination in the past 2 years

Table 4 also shows risk ratios for influenza vaccination by demographic characteristics. Among young adults and patients with diabetes, each added year of age was associated with a 1 percentage point improvement in vaccination, with opposite age effects for vaccination among older adults. For all 3 patient strata (young adults, older adults, patients with diabetes), vaccination rates this season were substantially higher if patients were vaccinated in prior seasons. Vaccination rates were higher among Asians, but lower among Black patients.

### Vaccination Rates Including Patient Self-Reported Data in Response to Portal Reminders

One-third (33%) of influenza vaccines were from outside UCLA Health (*n*=27,157). If including community-based influenza vaccinations self-reported in response to the patient portal reminders (Appendix 3a–c in the Supplementary Information), patients sent pre-commitment reminders did not have higher vaccination rates than controls, but patients sent either loss and gain frame reminders had higher rates than controls by 1–3 percentage points (adjusted risks 1.02 to 1.07). The largest effects were for young adults.

### Secondary Analyses

One possible reason for lack of impact is that only 12% of patients opened the pre-commitment portal messages sent in mid-October and 79%, 85%, and 62% opened the first, second, or third portal messages sent late October–December. Using the instrumental variable approach to evaluate effects on subgroups who opened the pre-commitment message, we estimate that the pre-commitment message raised vaccination rates by 4.5 percentage points (95% CI: −0.7 to +9.7) in the young adult group who viewed it, with no effect for older adults or diabetic patients. Using a similar instrumental variables approach, we did not find effects of loss/gain framing on patients who opened a portal reminder. Thus, even among patients who opened the portal reminders, their effect was minimal.

We evaluated whether the effects of the reminders or pre-commitment had differential effects within the 18–64-year age stratum. Tests for linear age interaction were not significant (*p*=0.35 for reminders, and *p*=0.46 for pre-commitment). We evaluated whether pre-commitment or portal reminders (loss/gain framing) increased influenza vaccination by 12/15/2019 (i.e., earlier than our primary end date of 03/31/2020); results were similar to those for the primary end date. Finally, we evaluated the interventions’ impact on patients who logged onto the portal more often than the median user in the prior year. The modest impact of pre-commitment reminders on young adults was limited to high portal users, but other findings did not change.

## DISCUSSION

In this randomized trial across a large health system in the year prior to the pandemic, patient portal reminders for influenza vaccinations that were tailored to patient characteristics (young adults, older adults, and diabetic patients) and incorporated two behavioral science strategies (pre-commitment and loss/gain framing) had no effect on raising influenza vaccination rates.

When we included additional self-reported vaccinations received outside the health system, both loss and gain framed messages among young adults and older adults had a modest impact with adjusted relative risks of vaccination (1.02 to 1.07) similar to other reminder/recall studies.^5^ It is possible that portal reminders stimulated vaccination at external sites (e.g., workplace) that did not merge with the EHR, possibly because such sites were convenient. However, we would have expected a dampening but not elimination of the intervention effects for the main analysis that excluded these self-reported vaccinations.

This intervention was not designed to address vaccine hesitancy, which we suspect is the major reason for the intervention’s small impact .^11,13,37–40^ Notably, during the same influenza season, our group found that for children, portal reminder messages had virtually no impact on first-dose influenza vaccination but did have a strong impact on receipt of second doses for which vaccine hesitancy is not an issue.^41^ Interventions to directly address hesitancy are needed.

We shaped the portal messages around findings from our MTurk survey in which both pre-commitment and message framing had positive effects on intent to vaccinate, yet these findings did not hold up in our pragmatic trial. More studies are needed to compare patients’ hypothetical versus real world responses.

Study strengths include a large, pragmatic clinical trial, randomization to account for many unmeasured provider or practice-level factors, and capture of influenza vaccinations without ascertainment bias. One limitation is potential lack of generalizability from one health system, particularly if vaccine hesitancy is particularly high in this health system. Also, we used broad age categories; messages tailored to more granular age categories might have larger impact. We selected patients with diabetes because they are at higher risk and are identifiable using SUPREME criteria; messages tailored to other chronic diseases might have an impact. Further to this, since we already had multiple study arms, we did not formally evaluate other psychological principles. Other limitations include inability to generalize to non-portal users and inability to identify all vaccinations received outside the health system despite very strong data linkages between our EHR and pharmacy and other databases.

We conclude that patient reminders sent by a health system’s patient portal that were tailored to patient characteristics (young adults, older adults, diabetes), and reminders that incorporated the behavioral science messaging strategies of pre-commitment and loss/gain framing, were not effective in raising pre-COVID influenza vaccination rates. Further studies to optimize patient reminders, including studies using alternative modalities such as text message reminders, are needed.

## Supplementary Information

ESM 1 (DOCX 40 kb)

ESM 2 (DOCX 13 kb)

ESM 3 (DOCX 34 kb)
