# Supplementary material for: Effect of Personalized Messages Sent by a Health System’s Patient Portal on Influenza Vaccination Rates: a Randomized Clinical Trial
Source: J Gen Intern Med. 2021 Sep 1;37(3):615–23. doi: 10.1007/s11606-021-07023-w (PMC8858355; doi:10.1007/s11606-021-07023-w)
Supplement: Supplementary file 1 — (DOCX 40 kb) [file 11606_2021_7023_MOESM1_ESM.docx]

**Appendix 1:**

**Methods and Results of Pre-Test of Messages**

The goal for this preliminary work was to identify promising psychological factors that might increase influenza vaccine uptake to include in our message to patients.

**Methods**

In September of 2019, we recruited 4317 participants from Amazon’s Mechanical Turk platform. We excluded anyone who failed an Instructional Manipulation Check similar to Oppenheimer et al. (2009), and were thus left with a sample of 3896 participants (Mage = 37.8, SDage=11.8). With an average of 177 participants per cell, we randomly assign participants to one of 22 study arms corresponding to our 2 (treatment message or control) x 11 (various psychological factors being tested) experimental design. The psychological hypotheses we tested were:

- **Gains versus losses** (e.g., Tversky & Kahneman, 1992): Framing a negative outcome as a loss has a larger negative psychological impact than the same outcome framed instead as a foregone gain.
- **Identity labeling** (e.g., Bryan et al., 2011): Associating a personal, aspirational identity with a behavior can induce label-consistent actions.
- **Provincial norms** (e.g., Goldstein et al., 2008): Informing people of a hyper-local descriptive social norm engenders conformity.
- **Prosocial motivation** (e.g., Li et al., 2016): People are motivated to receive vaccines when framed in terms of benefits to others not just themselves.
- **Omission bias** (e.g., Ritov & Baron, 1995): Bad outcomes are judged to be worse if they result from an action (i.e., a commission) rather than an inaction (i.e., an omission).
- **Reciprocity** (Falk & Fischbacher, 2006): People feel a desire to reciprocate in response to receiving benefits.
- **Scarcity** (e.g., Lynn, 1989): Benefits that are seen to be more scarce are valued more highly than benefits that are in unlimited supply.
- **Authority** (e.g., Cialdini, 1987): Messages from people who are seen as relevant authorities are more persuasive than messages from neutral sources.
- **Self-Efficacy** (e.g., Tannenbaum et al., 2015): Fear appeals are persuasive when coupled with messages of self-efficacy.
- **Self-Consistency** (Bem, 1972): People seek consistency with prior behavior and use past actions to guide future decisions.

Table 1 contains the control and treatment message for each of the 11 psychological factors.

| *Table 1. Control and Treatment messages used for all 11 psychological factors tested in the pre-test.* | | |
| --- | --- | --- |
| **Principle** | **Control Message** | **Treatment Message** |
| *gains v losses* | Getting your flu vaccine now may prevent a doctor’s visit for illness later. | Not getting your flu vaccine may lead to a doctor’s visit for illness later. |
| *gains v losses v2* | Vaccinating yourself against the flu this season is the best way to protect yourself from getting sick or being hospitalized from the flu. | Failing to get vaccinated against the flu this season increases your chances of getting sick or being hospitalized from the flu. |
| *behavior v identity labeling* | Take a healthy action! Get a flu vaccine as soon as possible. | Be a healthy person! Get a flu vaccine as soon as possible. |
| *global v provincial norms* | This year, most people in the U.S. will get a flu shot to stay well. Join them! | This year, most people in our healthcare system in your age group will get a flu shot to stay well. Join them! |
| *private v social costs* | Getting a flu vaccine now may stop you from contracting the flu. | Getting a flu vaccine now may stop you from spreading flu to your loved ones. |
| *omission v commission* | Failing to get the flu vaccine can lead to serious illness. | Choosing to not vaccinate can lead to serious illness. |
| *no reciprocity v reciprocity* | Others in your community have already gotten the flu vaccine this season. Will you get a vaccine to help keep others safe? | Others in your community have already gotten the flu vaccine this season, which helps to keep YOU safe and healthy. Will you get a vaccine to help keep others safe? |
| *no scarcity v scarcity* | Come get your flu vaccine ASAP! | Time is running out to maximize the benefit of your flu vaccine! Come get your flu vaccine ASAP before flu season peaks. |
| *no authority v authority* | It is strongly recommended that people should get the flu vaccine each year. Join them in preventing the flu. | Our healthcare system’s doctors and nurses—as well as the American College of Physicians—strongly recommend that people should get the flu vaccine each year. Join them in preventing the flu. |
| *no self-efficacy v self-efficacy* | Imagine yourself at home sick in December, in bed with a terrible flu. Do you want to schedule your flu vaccine today? | Imagine yourself at home sick in December, in bed with a terrible flu, thinking back to this very moment and wishing that you would’ve signed up for a flu vaccine right now. Do you want to schedule your flu vaccine today? |
| *no self-consistency v self-consistency* | Early indicators suggest this is going to be a bad flu season. Sign up for a flu vaccine today! | Haven’t gotten a flu vaccine before? That’s ok! This year, you have a reason to: Early indicators suggest this is going to be a bad flu season. Sign up for a flu vaccine today! |

Participants were instructed to imagine receiving the given message (depending on experimental condition) from their doctor’s office and then were asked six questions—the order of the first five questions was counterbalanced between participants—gauging their reaction to the message and their subsequent intent to vaccinate.

- **Likelihood:** What is the likelihood you would get the flu vaccine after receiving this message?
- **Affect**: How positively or negatively does this message make you feel about receiving a flu vaccine?
- **Motivation**: How motivated or demotivated does this message make you feel to receive a flu vaccine?
- **Persuasive**: How persuasive or unpersuasive about getting a flu vaccine did you find this message?
- **Recommend**ed: What is the likelihood that you would recommend to a friend that they get the flu vaccine after receiving this message?
- **Change:** If the message that we showed you was actually sent to you by your doctor's office, how would it change your intention to vaccinate?

Afterwards, we asked whether, prior to participating in the study, participants intended to get the flu vaccine as well as a host of demographic variables. We were primarily interested in three dependent measures: Likelihood, Change, and a Vaccination Index composed of the first five items listed above. These five measures showed sufficient reliability (Cronbach’s α = 0.93), so a simple mean was taken to construct the index of all five items.

**Results**

It is important to note that participants were reporting on *intent* to vaccinate in a hypothetical choice, and reports of hypothetical choice have been demonstrated to diverge frequently from actual behavior (e.g., Camerer & Mobbs, 2017). The purpose of this pre-test was simply to identify promising factors to incorporate into a standard messaging that all participants would receive in the full trial with the hopes of raising vaccine uptake as much as possible. Tables 2a, 2b, and 2c report on the effect of the treatment message (versus control) for all 11 psychological factors tested. In each column, we report the results of a linear regression predicting the three outcomes of interest, each on a 7-point scale, from a dummy variable corresponding to being in the Treatment condition, controlling for prior intent to vaccine and overall endorsement of vaccines.

Overall, we found evidence suggesting that the following factors may be especially potent to incorporate into messaging related to vaccination:

- **Gains framing:** Framing failure to vaccinate as a foregone gain (rather than a loss) significantly increased the *Change* item as well as the *Vaccination Index*.
- **Authority**: Sending the message from an authority significantly increased the *Change* item.
- **Scarcity**: Describing the chance to receive the optimal efficacy of the vaccine as scarce and dwindling significantly increased the *Likelihood* item and the *Vaccination Index*.
- **Omission**: Framing the failure to vaccinate as a commission, rather than an omission, significantly increased the *Likelihood* item.

While not definitive proof that these messages would be effective in motivating actual vaccine uptake, these data were suggestive enough for us to incorporate each of the principles into our message design.

| **Table 2a. Comparing All Manipulations** | | | | | | | | | | | |
| --- | --- | --- | --- | --- | --- | --- | --- | --- | --- | --- | --- |
|  | | | | | | | | | | | |
|  | Dependent variable: | | | | | | | | | | |
|  |  | | | | | | | | | | |
|  | Likelihood | | | | | | | | | | |
|  | loss | loss v2 | id label | prvncl norm | social cost | commission | reciprocity | scarcity | authority | self-efficacy | self-consistency |
|  | | | | | | | | | | | |
| condition | -0.038 | 0.0005 | 0.042 | 0.108 | 0.089 | 0.288^*^ | 0.044 | 0.425^***^ | -0.127 | 0.121 | -0.014 |
|  | (0.133) | (0.111) | (0.125) | (0.105) | (0.105) | (0.118) | (0.105) | (0.118) | (0.113) | (0.149) | (0.110) |
|  |  |  |  |  |  |  |  |  |  |  |  |
| prior_intent | 0.636^***^ | 0.760^***^ | 0.680^***^ | 0.716^***^ | 0.716^***^ | 0.633^***^ | 0.716^***^ | 0.713^***^ | 0.726^***^ | 0.668^***^ | 0.718^***^ |
|  | (0.035) | (0.027) | (0.031) | (0.027) | (0.028) | (0.031) | (0.027) | (0.029) | (0.028) | (0.038) | (0.029) |
|  |  |  |  |  |  |  |  |  |  |  |  |
| endorse | -1.094^***^ | -0.590^***^ | -0.826^***^ | -0.789^***^ | -0.630^***^ | -1.011^***^ | -0.918^***^ | -0.790^***^ | -0.995^***^ | -0.719^**^ | -1.004^***^ |
|  | (0.198) | (0.164) | (0.175) | (0.156) | (0.150) | (0.170) | (0.152) | (0.170) | (0.162) | (0.218) | (0.155) |
|  |  |  |  |  |  |  |  |  |  |  |  |
|  | | | | | | | | | | | |
| Observations | 355 | 352 | 362 | 356 | 361 | 365 | 354 | 347 | 355 | 343 | 346 |
| R^2^ | 0.672 | 0.772 | 0.689 | 0.760 | 0.771 | 0.697 | 0.782 | 0.736 | 0.761 | 0.617 | 0.781 |
| Adjusted R^2^ | 0.669 | 0.770 | 0.686 | 0.758 | 0.770 | 0.695 | 0.780 | 0.734 | 0.759 | 0.613 | 0.779 |
|  | | | | | | | | | | | |
| Note: | ^*^p<.05, ^**^p<.01, ^***^p<0.001  coefficient estimates and (standard error) | | | | | | | | | | |

| **Table 2b. Comparing All Promising Manipulations** | | | | | | | | | | | |
| --- | --- | --- | --- | --- | --- | --- | --- | --- | --- | --- | --- |
|  | | | | | | | | | | | |
|  | Dependent variable: | | | | | | | | | | |
|  |  | | | | | | | | | | |
|  | Change | | | | | | | | | | |
|  | loss | loss v2 | id label | prvncl norm | social cost | commission | reciprocity | scarcity | authority | self-efficacy | self-consistency |
|  | | | | | | | | | | | |
| condition | -0.239^*^ | 0.136 | 0.148 | -0.013 | 0.086 | -0.047 | -0.014 | 0.225 | 0.272^*^ | 0.040 | 0.010 |
|  | (0.118) | (0.110) | (0.112) | (0.111) | (0.112) | (0.118) | (0.116) | (0.120) | (0.124) | (0.134) | (0.113) |
|  |  |  |  |  |  |  |  |  |  |  |  |
| prior_intent | 0.238^***^ | 0.226^***^ | 0.186^***^ | 0.199^***^ | 0.243^***^ | 0.184^***^ | 0.259^***^ | 0.200^***^ | 0.160^***^ | 0.208^***^ | 0.264^***^ |
|  | (0.031) | (0.027) | (0.028) | (0.029) | (0.030) | (0.031) | (0.030) | (0.029) | (0.031) | (0.034) | (0.029) |
|  |  |  |  |  |  |  |  |  |  |  |  |
| endorse | -0.685^***^ | -0.394^*^ | -0.548^***^ | -0.545^**^ | -0.511^**^ | -0.881^***^ | -0.627^***^ | -0.648^***^ | -0.935^***^ | -0.754^***^ | -0.518^**^ |
|  | (0.175) | (0.163) | (0.158) | (0.165) | (0.160) | (0.170) | (0.167) | (0.172) | (0.177) | (0.196) | (0.158) |
|  |  |  |  |  |  |  |  |  |  |  |  |
|  | | | | | | | | | | | |
| Observations | 355 | 352 | 362 | 356 | 361 | 365 | 354 | 347 | 355 | 343 | 346 |
| R^2^ | 0.331 | 0.267 | 0.221 | 0.230 | 0.313 | 0.265 | 0.328 | 0.245 | 0.246 | 0.243 | 0.343 |
| Adjusted R^2^ | 0.325 | 0.260 | 0.215 | 0.224 | 0.307 | 0.259 | 0.322 | 0.239 | 0.240 | 0.236 | 0.337 |
|  | | | | | | | | | | | |
| Note: | ^*^p<.05, ^**^p<.01, ^***^p<0.001  coefficient estimates and (standard error) | | | | | | | | | | |

| **Table 2c. Comparing All Manipulations** | | | | | | | | | | | |
| --- | --- | --- | --- | --- | --- | --- | --- | --- | --- | --- | --- |
|  | | | | | | | | | | | |
|  | Dependent variable: | | | | | | | | | | |
|  |  | | | | | | | | | | |
|  | Vaccination Index | | | | | | | | | | |
|  | loss | loss v2 | id label | prvncl norm | social cost | commission | reciprocity | scarcity | authority | self-efficacy | self-consistency |
|  | | | | | | | | | | | |
| condition | -0.232^*^ | -0.034 | -0.038 | 0.163 | 0.152 | 0.149 | 0.085 | 0.364^***^ | 0.063 | 0.228 | -0.017 |
|  | (0.112) | (0.093) | (0.105) | (0.096) | (0.097) | (0.105) | (0.100) | (0.101) | (0.103) | (0.126) | (0.094) |
|  |  |  |  |  |  |  |  |  |  |  |  |
| prior_intent | 0.412^***^ | 0.509^***^ | 0.429^***^ | 0.448^***^ | 0.454^***^ | 0.408^***^ | 0.460^***^ | 0.457^***^ | 0.454^***^ | 0.409^***^ | 0.454^***^ |
|  | (0.029) | (0.023) | (0.026) | (0.025) | (0.025) | (0.027) | (0.026) | (0.025) | (0.025) | (0.032) | (0.025) |
|  |  |  |  |  |  |  |  |  |  |  |  |
| endorse | -0.844^***^ | -0.627^***^ | -0.774^***^ | -0.599^***^ | -0.759^***^ | -0.797^***^ | -0.858^***^ | -0.936^***^ | -0.850^***^ | -0.881^***^ | -0.843^***^ |
|  | (0.167) | (0.137) | (0.148) | (0.142) | (0.138) | (0.151) | (0.145) | (0.146) | (0.147) | (0.184) | (0.133) |
|  |  |  |  |  |  |  |  |  |  |  |  |
|  | | | | | | | | | | | |
| Observations | 355 | 352 | 362 | 356 | 361 | 365 | 354 | 347 | 355 | 343 | 346 |
| R^2^ | 0.572 | 0.703 | 0.582 | 0.611 | 0.661 | 0.566 | 0.647 | 0.652 | 0.628 | 0.513 | 0.683 |
| Adjusted R^2^ | 0.568 | 0.700 | 0.579 | 0.608 | 0.658 | 0.562 | 0.644 | 0.649 | 0.625 | 0.509 | 0.681 |
|  | | | | | | | | | | | |
| Note: | ^*^p<.05, ^**^p<.01, ^***^p<0.001  coefficient estimates and (standard error) | | | | | | | | | | |

We used these results to design our messages to patients in the main study.

**References for Appendix 1**

Bem, D. J. (1972). Self-perception theory. *Advances in Experimental Social Psychology*, *6*, 1–62.

Bryan, C. J., Walton, G. M., Rogers, T., & Dweck, C. S. (2011). Motivating voter turnout by invoking the self. *Proceedings of the National Academy of Sciences*, *108*(31), 12653–12656. https://doi.org/10.1073/pnas.1103343108

Camerer, C., & Mobbs, D. (2017). Differences in Behavior and Brain Activity during Hypothetical and Real Choices. *Trends in Cognitive Sciences*, *21*(1), 46–56. https://doi.org/10.1016/j.tics.2016.11.001

Cialdini, Robert B. (1987). *Influence* (Vol. 3). HarperCollins.

Falk, A., & Fischbacher, U. (2006). A theory of reciprocity. *Games and Economic Behavior*, *54*(2), 293–315. https://doi.org/10.1016/j.geb.2005.03.001

Goldstein, N. J., Cialdini, R. B., & Griskevicius, V. (2008). A room with a viewpoint: Using social norms to motivate environmental conservation in hotels. *Journal of Consumer Research*, *35*(3), 472–482. https://doi.org/10.1086/586910

Li, M., Taylor, E. G., Atkins, K. E., Chapman, G. B., & Galvani, A. P. (2016). Stimulating Influenza Vaccination via Prosocial Motives. *PLOS ONE*, *11*(7), e0159780. https://doi.org/10.1371/journal.pone.0159780

Lynn, M. (1989). Scarcity effects on desirability: Mediated by assumed expensiveness? *Journal of Economic Psychology*, *10*(2), 257–274. https://doi.org/10.1016/0167-4870(89)90023-8

Oppenheimer, D. M., Meyvis, T., & Davidenko, N. (2009). Instructional manipulation checks: Detecting satisficing to increase statistical power. *Journal of Experimental Social Psychology*, *45*, 867–872. https://doi.org/10.1016/j.jesp.2009.03.009

Ritov, I., & Baron, J. (1995). Outcome Knowledge, Regret, and Omission Bias. *Organizational Behavior and Human Decision Processes*, *64*(2), 119–127.

Tannenbaum, M. B., Hepler, J., Zimmerman, R. S., Saul, L., Jacobs, S., Wilson, K., & Albarracin, D. (2015). Appealing to fear: A Meta-Analysis of Fear Appeal Effectiveness and Theories. *Psychological Bulletin*, *141*(6), 1178–1204. https://doi.org/10.1037/a0039729

Tversky, A., & Kahneman, D. (1992). Advances in prospect theory: Cumulative representation of uncertainty. *Journal of Risk and Uncertainty*, *5*(4), 297–323.
