# Supplementary material for: Effect of Personalized Messages Sent by a Health System’s Patient Portal on Influenza Vaccination Rates: a Randomized Clinical Trial
Source: J Gen Intern Med. 2021 Sep 1;37(3):615–23. doi: 10.1007/s11606-021-07023-w (PMC8858355; doi:10.1007/s11606-021-07023-w)
Supplement: Supplementary file 2 — (DOCX 13 kb) [file 11606_2021_7023_MOESM2_ESM.docx]

**Appendix 2.** Text of the Pre-Commitment Letter (mid-October) and the late-October Reminder Letter for gain-frame and loss-frame, for adults >65 years.

| Pre-Commitment Letter  Question to Recipient | The flu season is nearing and we are interested in knowing your plans regarding the flu vaccine. Please let us know by clicking the Flu Season Question above.  We recommend that you get your flu vaccine early. Take action today, such as scheduling a visit to your doctor’s office, or adding a reminder to your calendar.  Thank you for providing this information!  **Question: Are you planning to get the flu vaccine this season? (Yes/No/Not sure)** |
| --- | --- |
| Adult Reminder Letter  Gain Frame | Time is running out to maximize the benefit of your flu vaccine. **Call your doctor to set up an appointment ASAP, before flu season arrives.**  UCLA doctors and the American College of Physicians strongly recommend the flu vaccine each year.  **Choosing to get vaccinated** this season increases your chance of **staying healthy** by preventing a serious illness or having a hospitalization from the flu.  Call us to make an appointment or click here to request an appointment online. |
| Adult Reminder Letter  Loss Frame | Time is running out to maximize the benefit of your flu vaccine. Call your doctor to set up an appointment ASAP, before flu season arrives.  UCLA doctors and the American College of Physicians strongly recommend the flu vaccine each year.  **Choosing not to get vaccinated** can lead to **serious illness**, worsened chronic health problems, and hospitalization due to the flu.  Call us to make an appointment or click here to request an appointment online. |
| Older Adult Reminder Letter  Loss Frame | Time is running out to maximize the benefit of your flu vaccine. **Call your doctor to set up an appointment ASAP, before flu season arrives.**  UCLA doctors and the American College of Physicians strongly recommend the flu vaccine each year for persons age 65 and older. Remember, Medicare pays for the flu shot.  **Choosing not to get vaccinated** can lead to **serious illness**, worsened chronic health problems, and hospitalization due to the flu, especially for patients over the age of 65.  Call us to make an appointment or click here to request an appointment online. |
| Older Adult Reminder Letter  Gain Frame | Time is running out to maximize the benefit of your flu vaccine. **Call your doctor to set up an appointment ASAP, before flu season arrives.**  UCLA doctors and the American College of Physicians strongly recommend the flu vaccine each year for persons age 65 and older. Remember, Medicare pays for the flu shot.  **Choosing to get vaccinated** this season increases your chance of **staying healthy** by preventing a serious illness or having a hospitalization from the flu.  Call us to make an appointment or click here to request an appointment online. |
