# Supplementary material for: Effect of Personalized Messages Sent by a Health System’s Patient Portal on Influenza Vaccination Rates: a Randomized Clinical Trial
Source: J Gen Intern Med. 2021 Sep 1;37(3):615–23. doi: 10.1007/s11606-021-07023-w (PMC8858355; doi:10.1007/s11606-021-07023-w)
Supplement: Supplementary file 3 — (DOCX 34 kb) [file 11606_2021_7023_MOESM3_ESM.docx]

**Appendix 3.** Influenza vaccination rates including vaccinations self-reported by patients in response to the portal influenza reminders.

**Appendix 3a.** Influenza vaccination rates within patient strata (young adults 18-64 years, adults > 65 years, and adults with diabetes) and by pre-commitment (none or a pre-commitment message). These results include vaccinations self-reported by patients in response to the portal influenza reminders.

|  | **Young Adults 18-64yr**  **Without Diabetes** | | **Older Adults >65yr**  **Without Diabetes** | | **Adults with Diabetes** | |
| --- | --- | --- | --- | --- | --- | --- |
| **Pre-Commitment (No/Yes)** | **No** | **Yes** | **No** | **Yes** | **No** | **Yes** |
| **All Patients** | 38.6% | 39.0% | 56.8% | 57.6% | 61.9% | 62.6% |
| **Gender** |  |  |  |  |  |  |
| Female | 39.6% | 39.7% | 57.2% | 57.8% | 61.5% | 62.7% |
| Male | 37.2% | 38.0% | 56.3% | 57.3% | 62.3% | 62.5% |
| **Primary Insurer** |  |  |  |  |  |  |
| Private | 38.7% | 39.1% | 55.3% | 56.5% | 57.9% | 59.0% |
| Public | 39.1% | 39.4% | 57.8% | 58.5% | 71.4% | 70.5% |
| Other/Unknown | 32.6% | 34.6% | 65.4% | 55.6% | 51.7% | 65.6% |
| **Race** |  |  |  |  |  |  |
| White | 40.3% | 40.8% | 57.4% | 58.1% | 63.9% | 65.3% |
| Black | 29.3% | 30.8% | 46.2% | 47.8% | 53.3% | **50.3% ^a^** |
| Asian | 47.8% | 47.6% | 62.5% | 63.0% | 69.1% | 69.2% |
| Other/Multiple/Unknown | 33.9% | 34.5% | 53.9% | 54.9% | 55.5% | 56.0% |
| **Ethnicity** |  |  |  |  |  |  |
| Hispanic | 36.9% | 37.2% | 51.6% | 52.7% | 59.2% | 59.9% |
| Non-Hisp./Unknown | 38.8% | 39.2% | 57.1% | 57.8% | 62.3% | 63.0% |
| **Vaccine History** |  |  |  |  |  |  |
| None | 17.2% | 16.9% | 17.9% | 19.4% | 19.8% | **22.1%^a^** |
| Prior Vaccination^b^ | 60.8% | **61.9%^a^** | 70.0% | 69.9% | 79.0% | 78.4% |

**^a^**p<.05 ^b^Prior influenza vaccination in the past 2 years

|  | **Young Adults 18-64yr**  **Without Diabetes** | | | **Older Adults >65yr**  **Without Diabetes** | | | **Adults With Diabetes** | | |
| --- | --- | --- | --- | --- | --- | --- | --- | --- | --- |
|  | **None** | **Loss Frame** | **Gain Frame** | **None** | **Loss Frame** | **Gain Frame** | **None** | **Loss Frame** | **Gain Frame** |
| **All Patients** | 37.2% | 39.9% | 39.3% | 56.4% | 57.4% | 57.8% | 61.4% | 62.7% | 62.7% |
| **Gender** |  |  |  |  |  |  |  |  |  |
| Female | 37.6% | 40.8% | 40.4% | 56.2% | 57.8% | 58.4% | 61.2% | 62.2% | 62.8% |
| Male | 36.6% | 38.6% | 37.7% | 56.6% | 56.7% | 57.0% | 61.5% | 63.2% | 62.6% |
| **Primary Insurer** |  |  |  |  |  |  |  |  |  |
| Private | 37.3% | 40.0% | 39.4% | 54.2% | 56.8% | 56.6% | 57.4% | 58.7% | 59.1% |
| Public | 38.4% | 40.7% | 38.7% | 58.1% | 57.8% | 58.5% | 70.6% | 71.6% | 70.7% |
| Other/Unknown | 30.9% | 36.9% | 33.1% | 57.0% | 56.8% | 67.8% | 52.8% | 60.2% | 61.9% |
| **Race** |  |  |  |  |  |  |  |  |  |
| White | 38.7% | 41.7% | 41.3% | 56.8% | 58.2% | 58.2% | 63.1% | 65.5% | 65.2% |
| Black | 27.0% | **31.4%^a^** | 31.7% | 42.8% | 49.3% | 48.7% | 53.7% | 50.2% | 51.6% |
| Asian | 46.5% | 48.0% | 48.7% | 61.5% | 62.5% | 64.4% | 68.6% | 70.4% | 68.4% |
| Other/Multiple/Unknown | 33.0% | 35.5% | **33.9%^a^** | 54.9% | 53.1% | 55.1% | 54.9% | 55.5% | 56.8% |
| **Ethnicity** |  |  |  |  |  |  |  |  |  |
| Hispanic | 36.6% | 38.6% | **35.9% ^a^** | 50.3% | 54.3% | 51.6% | 60.7% | **58.3%^a^** | 59.7% |
| Non-Hisp./Unknown | 37.3% | 40.1% | 39.7% | 56.7% | 57.5% | 58.2% | 61.5% | 63.3% | 63.2% |
| **Vaccine History** |  |  |  |  |  |  |  |  |  |
| None | 16.1% | 17.6% | 17.5% | 17.7% | 19.5% | 18.5% | 19.6% | 22.6% | 20.6% |
| Prior Vaccination^b^ | 59.2% | 62.9% | **62.0%^a^** | 68.9% | 70.5% | 78.0% | 77.5% | 79.2% | 79.0% |
| **^a^**p<.05 ^b^Prior influenza vaccination in the past 2 years | | | | | | | | | |

**Appendix 3b:** Influenza vaccination rates by adult strata (adults 18-64 years, adults > 65 years, and adults with diabetes) and by reminder framing (none, loss-frame, or gain-frame). Results include vaccinations self-reported by patients in response to the portal influenza reminders.

**Appendix 3c.** Risk ratios (95% Confidence Intervals)^a^ from unadjusted and adjusted analyses, comparing loss/gain frame, pre-commitment (no/yes), and demographic characteristics within each of the 3 strata (young adults 18-64 years (without diabetes), older adults >65 years (without diabetes), and adults with diabetes. These results include vaccinations self-reported by

|  | **Young Adults Without Diabetes** | | **Older Adults Without Diabetes** | | **Adults With Diabetes** | |
| --- | --- | --- | --- | --- | --- | --- |
| **Study Arms and** | **Unadjusted** | **Adjusted** | **Unadjusted** | **Adjusted** | **Unadjusted** | **Adjusted** |
| **Sub-Groups** | **Risk Ratio** | **Risk Ratio** | **Risk Ratio** | **Risk Ratio** | **Risk Ratio** | **Risk Ratio** |
| **Pre-Commitment Arm**  *(Ref = None)* |  |  |  |  |  |  |
| Pre-commitment message | 1.01  (1.00, 1.03) | 1.01  (1.00, 1.02) | 1.01  (0.99, 1.03) | 1.01  (0.99, 1.02) | 1.01  (0.99, 1.03) | 1.00  (0.98, 1.02) |
| **Reminder Arm**  *(Ref = No Reminders)* |  |  |  |  |  |  |
| Loss Frame | **1.07**  **(1.05, 1.09)** | **1.07**  **(1.05, 1.08)** | 1.02  (0.99, 1.05) | **1.03**  **(1.01, 1.05)** | 1.02  (1.00, 1.05) | 1.03  (1.00, 1.05) |
| Gain Frame | **1.06**  **(1.04, 1.07)** | **1.06**  **(1.04, 1.07)** | 1.03  (1.00, 1.05) | 1.03  (1.00, 1.05)) | 1.02  (1.00, 1.05) | 1.03  (1.00, 1.04) |
|  |  |  |  |  |  |  |
| **Age (Each 1y older)** | **1.01**  **(1.01, 1.01)** | **1.01**  **(1.01, 1.01)** | 1.00  (1.00, 1.00) | **0.99**  **(0.99, 0.99)** | **1.01**  **(1.01, 1.01)** | **1.01**  **(1.00, 1.01)** |
| **Gender**  *(Ref = Male)* |  |  |  |  |  |  |
| Female | **1.03**  **(1.01, 1.05)** | **1.04**  **(1.02, 1.05)** | 1.01  (0.99, 1.04) | **1.03**  **(1.01, 1.06)** | **0.98**  **(0.96, 1.01)** | 1.01  (0.99, 1.03) |
| **Primary Insurer**  *(Ref = Private)* |  |  |  |  |  |  |
| Public | 0.99  (0.93, 1.04) | 0.98  (0.93, 1.03) | **1.04**  **(1.02, 1.06)** | **1.04**  **(1.02, 1.06)** | **1.20**  **(1.17, 1.23)** | 1.00  (0.99, 1.01) |
| Other/Unknown | **0.84**  **(0.76, 0.93)** | 0.92  (0.86, 0.99) | 1.06  (0.98, 1.14) | 1.06  (0.99, 1.14) | 0.98  (0.91, 1.06) | 0.97  (0.91, 1.02) |
| **Race**  *(Ref = White)* |  |  |  |  |  |  |
| Black/African-American | **0.72**  **(0.69, 0.76)** | **0.84**  **(0.81, 0.88)** | **0.83**  **(0.79, 0.87)** | **0.91**  **(0.86, 0.96)** | **0.79**  **(0.75, 0.83)** | **0.90**  **(0.87, 0.93)** |
| Asian | **1.12**  **(1.09, 1.15)** | **1.07**  **(1.05, 1.09)** | **1.09**  **(1.06, 1.12)** | **1.07**  **(1.04, 1.10)** | **1.04**  **(1.01, 1.08)** | 1.03  (1.00, 1.06) |
| Other/Multiple Races/ Unk | **0.83**  **(0.81, 0.85)** | **0.93**  **(0.91, 0.95)** | **0.93**  **(0.90, 0.96)** | 1.00  (0.97, 1.02) | **0.86**  **(0.83, 0.89)** | **0.95**  **(0.93, 0.97)** |
| **Ethnicity**  *(Ref = Non-Hisp/Other/ Unk)* |  |  |  |  |  |  |
| Hispanic or Latinx | **0.95**  **(0.91, 0.98)** | 1.00  (0.97, 1.02) | **0.91**  **(0.86, 0.96)** | **0.92**  **(0.87, 0.96)** | **0.95**  **(0.91, 0.98)** | 1.01  (0.98, 1.04) |
| **Vaccine History**  *(Ref = None)* |  |  |  |  |  |  |
| Prior Vaccination^b^ | **3.55**  **(3.42, 3.68)** | **3.46**  **(3.34, 3.59)** | **3.78**  **(3.55, 4.01)** | **3.79**  **(3.57, 4.03)** | **3.76**  **(3.56, 3.97)** | **3.61**  **(3.42, 3.82)** |
| ^a^The p-values for pre-commitment message and loss or gain frame use a significance threshold of 0.017; the rest use 0.05. All boldfaced cells have p<0.01 ^b^Prior influenza vaccination in the past 2 years | | | | | | |

patients in response to the portal influenza reminders.
